# Decolorization of the azo dye Acid Orange 51 by laccase produced in solid culture of a newly isolated *Trametes trogii* strain

**DOI:** 10.1007/s13205-012-0076-2

**Published:** 2012-07-17

**Authors:** Dalel Daâssi, Hela Zouari-Mechichi, Fakher Frikha, Maria Jesus Martinez, Moncef Nasri, Tahar Mechichi

**Affiliations:** 1 Laboratory of Enzyme Engineering and Microbiology, Ecole Nationale d’Ingénieurs de Sfax, University of Sfax, Route de Soukra Km 4,5, BP 1173, 3038 Sfax, Tunisia; 2 Centro de Investigaciones Biologicas, CSIC, Ramiro de Maeztu 9, 28040 Madrid, Spain

**Keywords:** Crude laccase, Synthetic textile dyes, Mediators, Optimization, Box–Behnken, Decolorization, Detoxification

## Abstract

This study concerns the decolorization and detoxification of the azo dye Acid Orange 51 (AO51) by crude laccase from *Trametes trogii* produced in solid culture using sawdust as support media. A three-level Box–Behnken factorial design with four factors (enzyme concentration, 1-hydroxybenzotriazole (HBT) concentration, dye concentration and reaction time) combined with response surface methodology was applied to optimize AO51 decolorization. A mathematical model was developed showing the effect of each factor and their interactions on color removal. The model predicted that Acid Orange 51 decolorization above 87.87 ± 1.27 % could be obtained when enzyme concentration, HBT concentration, dye concentration and reaction time were set at 1 U/mL, 0.75 mM, 60 mg/L and 2 days, respectively. The experimental values were in good agreement with the predicted ones and the models were highly significant, the correlation coefficient (*R*^2^) being 0.9. Then the desirability function was employed to determine the optimal decolorization condition for each dye and minimize the process cost simultaneously. In addition, germination index assay showed that laccase-treated dye was detoxified; however in the presence of HBT, the phytotoxicity of the treated dye was increased. By using cheap agro-industrial wastes, such as sawdust, a potential laccase was obtained. The low cost of laccase production may further broaden its application in textile wastewater treatment.

**Electronic supplementary material:**

The online version of this article (doi:10.1007/s13205-012-0076-2) contains supplementary material, which is available to authorized users.

## Introduction

Progress in industrialization, in particular textile industries, has led to the discharge of unprecedented amount of wastewater containing synthetic dyes, which can be a source of dangerous by-products from oxidation, hydrolysis or other chemical reactions in the wastewater solution.

Azo dyes are the most important group of synthetic colorants. They are generally considered as xenobiotic compounds that are very recalcitrant against biodegradative processes. Several azo dyes and their degradation products might be mutagenic and/or carcinogenic to microorganisms, aquatic life and human beings (Oh et al. 1997; de Aragao Umbuzeiro et al. 2005; Saratale et al. 2011).

Many studies have indicated that aromatic amines that arise from the azo reduction and cleavage of azo dyes are thought to be activated as mutagens through their N-oxidation by cytochrome P450 isozymes (Chung 2000). Recently, in the food industry some azo dyes, such as orthonitroaniline orange and amaranth (FDS-c red number 5) (Tucson University: “Health and Safety in the Arts, A Searchable Database of Health and Safety Information for Artists”), have been banned due to toxic side effects (potential chronic toxicity).

Toxicity, mutagenicity and carcinogenicity of azo dyes are, therefore, of major concern from the human health point, particularly with occupational groups such as dyestuff and textile dyers. These workers have been reported to have incidences of bladder cancer (Chung and Stevens 1992).

Thus, environmental legislation has imposed stringent limits on the concentrations of dye pollutants as chemical oxygen demand (COD) and biochemical oxygen demand (BOD). For example, the limits of BOD and COD in the industrial effluents from dyeing/textile industries have been set at 30 and 100–160 mg/dm^3^, respectively, according to the Effluent Standard in Taiwan (Taiwan Environmental Protection Administration (EPA): effluent standards. http://law.epa.gov.tw/en/laws/480770486.html).

Hence, the removal of dyes from colored effluents is essential, and various physical, chemical and biological dye removal techniques from aqueous solutions have been developed (Crini and Badot 2008). Conventional chemical and physical discoloration processes, including adsorption (Pedro Silva et al. 2004; Thinakaran et al. 2008), coagulation (Golob et al. 2005; Bali and Karagozoglu 2007), filtration (Capar et al. 2006), ozonation (Muthukumar and Selvakumar 2006), photo-fenton process (Ilha et al. 2009), UV/NaOCl (Zeng et al. 2009), electrochemical oxidation (Soloman et al. 2009), ultrasonic irradiation (Madhavan et al. 2010) and UV/H_2_O_2_ (Damle and Shukla 2010) have also been utilized.

Nevertheless, these treatment processes enable only the separation of dyes without degrading them, thereby producing large quantities of sludge and creating waste disposal problems. A secondary pollution problem may arise from excessive chemical use.

In recent years, biological decolorization techniques have been considered as alternative, environmentally friendly methods for dye detoxification and color removal. Enzyme methods applied in dye degradation have low energy costs, are easy to control and have low impacts on ecosystems. Recent studies have shown that fungi or enzymes from fungi are able to decolorize and detoxify industrial dyes (Cristóvão et al. 2008; Maalej-Kammoun et al. 2009). Decolorization of dye wastewater by the action of the enzyme laccase has been the subject of many studies (Mechichi et al. 2006; Khlifi et al. 2010; Neifar et al. 2011).

In the current work, we studied the high potential applications of laccase produced in solid culture under sawdust of a newly isolated *Trametes trogii* strain in the decolorization and detoxification of textile dye. Acid Orange 51 (AO51: CAS 8003-88-1) (Table 1) is a water-soluble anionic azo dye with typically one to three sulfonic groups, which is widely applied to wool, silk and polyamide. The toxic nature of the dye is still not quantified much, but the sulfonated azo dyes may be composed of naphthalene sulfonic acids, naphthols, naphthoic acids, benzidines, etc. Benzidine-based azo dyes are in focus because of the carcinogenicity of benzidine. Like many other dyes of its class, AO51 is bright in color due to the presence of one or several azo (–N=N–) groups associated with substituted aromatic structures. Many studies have considered physical adsorption techniques as the preferred means for removing acid dyes (Tsai et al. 2004; Pedro Silva et al. 2004), whereas the decolorization of AO51 by the action of laccase has been the subject of few studies.

**Table 1:** Some properties of the Acid Orange 51 used in the present study

Property	Acid Orange 51
CAS	8003-88-1
C.I.	26550
Chemical formula	C_36_H_26_O_11_N_6_S_3_Na_2_
Molecular weight	860.81
Molecular size	2.96 × 0.92 × 0.90 nm
Dye content	∼50 %
λ_max_ (nm)	450
Molecular structure	

Purchased from Aldrich Co., USA

Incubation time, and initial enzyme, dye and HBT concentrations are generally the most important parameters that significantly influence the enzymatic degradation process. Since the conventional method of optimization, “one factor at a time” approach is laborious, time consuming and incomplete, response surface methodology (RSM) using Box–Behnken (as factorial experimental design) was applied to model the decolorization process, to identify possible interactions and determine the optimum operational conditions.

The application of RSM in the textile effluent treatment process can result in improved decolorization and reduced process variability, time and overall costs. In addition, the factors that influence the degradation processes can be identified and optimized, and possible synergistic or antagonistic interactions that may exist between factors can be evaluated (Box and Behnken 1978). Several studies have been published recently on the application of RSM in color removal optimization by enzymatic catalysis (Murugesan et al. 2007; Neifar et al. 2011). The most common and efficient design used in response surface modeling is the Box–Behnken design. Compared to the central composite and Doehlert designs, Box–Behnken presents some advantages such as requiring few experimental points for its application (three levels per factor) and high efficiency (Costa Ferreira et al. 2007).

## Materials and methods

### Chemicals and instruments

The AO51 dye used in the present study was purchased from Aldrich Chemical Co. (St. Louis, USA). The identification information and molecular structure of this dye is depicted in Table 1. 2,6-Dimethoxyphenol (DMP), 1-hydroxybenzotriazole (HBT), *m*-, *o*- and *p*-coumaric acid, acetosyringone and Tempo were obtained from Aldrich Chemical Co. (St. Louis, USA).

All pH measurements were performed using a pH meter, Model 744 (Metrohm Instruments, Suisse). Absorbance measurements were carried out with UV–visible spectrophotometer (Shimadzu UV 1650 PC).

### Fungal strain, and media and culture conditions

The fungal isolate used in this study was *T*. *trogii.* The isolate was maintained in the culture collection of our laboratory (Maalej-Kammoun et al. 2009). For short-term conservation, the isolate was maintained on 2 % malt extract and 1 % agar. Petri dishes were cultured at 30 °C and stored at 4 °C.

The crude laccase was produced under solid-state fermentation (SSF) conditions by *T*. *trogii.* In 250-mL Erlenmeyer flasks containing 5 g of sawdust, 25 mL of culture medium contained glucose (20 g/L), casein peptone (4.5 g/L), ratio solid/liquid (5 g/L), MgSO_4_ (0.5 g/L), KH_2_PO_4_ (0.05 g/L) and trace element solution (1 mL). The trace element solution composition was as follows (g/L): B_4_O_7_Na_2_·10H_2_O, 0.1; CuSO_4_·5H_2_O, 0.01; FeSO_4_·7H_2_O, 0.05; MnSO_4_·7H_2_O, 0.01; ZnSO_4_·7H_2_O, 0.07; (NH4)_6_Mo_7_O_24_·4H_2_O, 0.01. The pH of the solution was adjusted to 5.5. The medium was sterilized at 121 °C for 20 min.

Inoculation was carried out directly in the Erlenmeyer flasks. Six agar plugs (diameter, 6 mm), from an actively growing fungus on malt extract (ME) per Erlenmeyer, were used as inoculum. The flasks were capped with cotton stoppers, which permitted passive aeration, and were incubated at 30 °C under dark. Extracellular enzymes from SSF were extracted with phosphate buffer (100 mM, pH 7.0) (10 mL buffer/g substrate), by shaking for 1 h at 160 rpm at room temperature.

### Determination of laccase activity and properties

Laccase activity was measured by monitoring the increase in absorbance at 469 nm (ε_469nm_ = 27,500/M/cm) of a reaction mixture containing 10 mM 2,6-dimethoxyphenol in 100 mM acetate buffer, pH 5.0. Enzymatic reactions were carried out at room temperature. One unit of enzyme activity was defined as the amount of enzyme oxidizing 1 μmol of substrate per minute.

### Decolorization tests

Decolorization of AO51 was examined using the crude *T. trogii* laccase preparation produced in solid culture using sawdust. All experiments in Box–Behnken design were performed using 50-mL disposable flasks in a 5-mL final reaction volume. The reaction mixture, containing 100 mM acetate buffer pH 5.0, different concentrations of HBT, dye (20–100 mg/L) and laccase from culture filtrate (0.5–1.5 U/mL), was incubated in the dark (30 °C). These reactions were prepared as shown in Table 2 according to the experimental design (Table 3): different concentrations of enzyme (0.5, 1 and 1.5 U/mL), HBT (0.5, 0.75 and 1 mM) and dye (20, 60, 100 mg/L), pH (3.0, 4.5 and 6.0) and incubation times (1, 2 and 3 days) were used as variables.

**Table 2:** Range of variables used in the experimental design

	Variables	Units	Levels		
			−1	0	1
*X*_1_	Enzyme concentration	U/mL	0.5	1	1.5
*X*_2_	HBT concentration	mM	0.5	0.75	1
*X*_3_	Dye concentration	mg/L	20	60	100
*X*_4_	Incubation time	Days	1	2	3

**Table 3:** Experimental conditions in the Box–Behnken design and the corresponding experimental responses

Run	Enzyme	HBT	Dye	Time	Decolorization (%)	
					Actual value	Predicted value
1	−1	−1	0	0	45.88	54.25
2	1	−1	0	0	57.87	54.41
3	−1	1	0	0	70.05	75.03
4	1	1	0	0	81.92	75.07
5	0	0	−1	−1	31.02	33.88
6	0	0	1	−1	47.21	43.40
7	0	0	−1	1	54.83	60.16
8	0	0	1	1	52.76	51.42
9	−1	0	0	−1	53.62	58.64
10	1	0	0	−1	37.82	40.17
11	−1	0	0	1	58.43	57.23
12	1	0	0	1	79.75	75.88
13	0	−1	−1	0	39.87	41.17
14	0	1	−1	0	59.01	66.29
15	0	−1	1	0	52.09	45.96
16	0	1	1	0	62.43	62.28
17	−1	0	−1	0	68.77	53.13
18	1	0	−1	0	58.98	57.84
19	−1	0	1	0	59.67	58.14
20	1	0	1	0	40.65	53.62
21	0	−1	0	−1	32.05	30.14
22	0	1	0	−1	69.65	65.15
23	0	−1	0	1	59.76	61.59
24	0	1	0	1	68.76	68.01
25	0	0	0	0	85.43	86.60
26	0	0	0	0	87.98	86.60
27	0	0	0	0	84.85	86.60
28	0	0	0	0	87.87	86.60
29	0	0	0	0	86.87	86.60

A reaction mixture without enzyme was prepared under the same conditions to detect possible color changes not due to enzyme activity. Controls contained heat-killed enzymes, whereas blanks used all components of the reaction mixture except the textile dye. All experiments were performed in duplicate.

Decreases in the absorbance maxima characteristics of AO51 (450 nm) were measured at different incubation times, and the percentage of dye decolorization was calculated from these data. Absorption spectra between 300 and 800 nm were recorded during dye decolorization with or without mediator by use of UV–visible spectrophotometer (Shimadzu UV 1650 PC). In parallel, control samples were maintained without enzyme under similar conditions.

### Effect of laccase mediators on dyes decolorization

The effect of phenolic and nonphenolic compounds as laccase mediators on AO51 decolorization was tested with HBT, acetosyringone, *o*-, *p*- and *m*-coumaric acids and Tempo. All compounds were used at 1 mM concentration in the reaction mixture, along with 50 mg/L dye concentration in 100 mM acetate buffer, pH 5.0. The reaction was initiated by the addition of 2 U/mL laccase and further incubated for 6 h in the dark at 30 °C.

### Effect of pH and temperature on dye decolorization by the laccase–HBT system

To study the effect of pH on textile dye decolorization, the reaction mixture containing 50 mg/L was incubated at 30 °C in the presence of 1 U/mL crude laccase and 0.5 mM HBT at different pH values using 100 mM acetate buffer of pH 4.0 and 5.0, and 100 mM phosphate buffer of pH 6.0, 7.0 and 8.0. The effect of temperature was examined by incubating 60 mg/L dye concentration in 100 mM acetate buffer of pH 5.0, 1 mM HBT and in the presence of 1 U/mL laccase at 30, 40, 50 and 60 °C. For each pH and temperature value, a reaction mixture without enzyme was prepared under the same conditions to detect possible color changes not due to enzyme activity. Controls contained heat-killed enzymes, whereas blanks used all components of the reaction mixture except the textile dye.

### Box–Behnken designs and response surface analysis

RSM using Box–Behnken was employed to optimize the four selected factors [(*X*_1_ enzyme concentration (U/mL), *X*_2_ HBT concentration (mM), *X*_3_ dye concentration (mg/L), *X*_4_ incubation time (days)] for enhancing the decolorization yield of Acid Orange 51. The four independent factors were investigated at three different levels (−1, 0, +1) (Table 2) and the experimental design used for study is shown in Table 3.

The decolorization yield of the three selected dyes was fitted using a second-order polynomial equation and a multiple regression of the data was carried out for obtaining an empirical model related to the factors. The general form of the second-order polynomial is:1where *Y* is the predicted response, *X*_*i*_ and *X*_*j*_ are independent factors, β_0_ is the intercept, β_*i*_ is the linear coefficient, β_*ii*_ is the quadratic coefficient and β_*ij*_ is the interaction coefficient.

Design-Expert, version 7.0 (STAT-EASE Inc., Minneapolis, USA) was used for the experimental designs and statistical analysis of the experimental data. The analysis of variance (ANOVA) was used to estimate the statistical parameters.

### Germination test

For the germination test, cress seeds (*Lepidium sativum* L.) were used. Fifteen seeds were placed on five layers of filter paper (Schleicher and Schuell no. 595, 85-mm round filter) in 9-cm Petri dishes and 5 mL of each decolorization reaction. Petri dishes were prepared under the same conditions as negatives controls (T1: enzyme with HBT, T2: enzyme without HBT and T3: heat-killed enzymes) to detect possible phytotoxicity due not to the dye. Distilled water was used as a control. The experiment had a complete randomized block design with three blocks and two pseudo-replications (i.e., two Petri dishes with the same dilution). The Petri dishes were incubated in a growth chamber at 25 °C. At 72 h after the beginning of the incubation, the percentage of germination was recorded. A visible root was used as the operational definition of seed germination. After 72 h, also the length of the roots was measured.

The percentages of relative seed germination (RSG) after 24, 48 and 72 h, and relative root growth (RRG) and germination index (GI) after 72 h of decolorized dyes were calculated as follows:

## Results and discussions

### Screening for laccase mediators

**Fig. 1:**
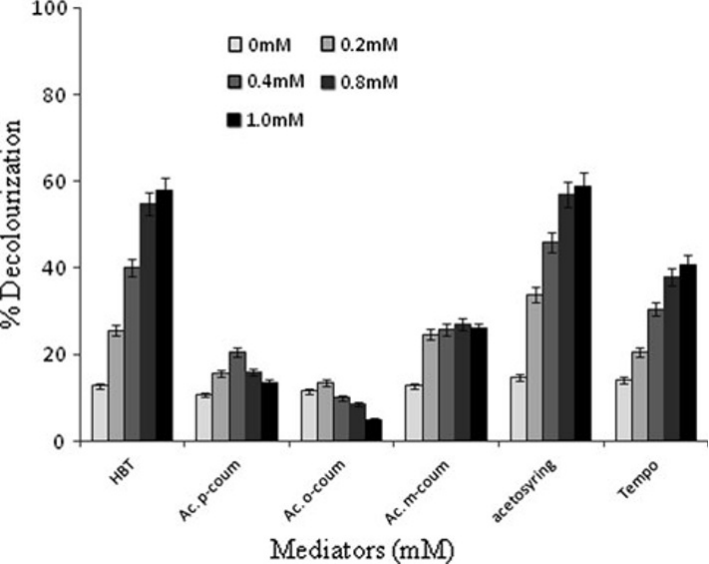
Effect of laccase mediators on the decolorization of Acid Orange 51 by crude*T*. *trogii* laccase: 1-hydroxybenzotriazole (HBT), acetosyringone (acetosyring), acid *p*-coumaric (Ac. *p*-coum), *o*-coumaric (Ac. *o*-coum), *m*-coumaric (Ac. *m*-coum) and Tempo

Figure 1 shows that among the six compounds (acetosyringone, HBT, Tempo, *p*- *o*- and *m*-coumaric acids) screened as laccase mediators at 1 mM concentration, acetosyringone and HBT produced the strongest decolorization rate, >50 % in 6 h. In addition, the decolorization of Acid Orange increased with acetosyringone and HBT concentrations. However, the rate of decolorization in the presence of *o*-, *m*- and *p*-coumaric acid was inversely proportional to the mediator concentration.

It can be seen also that at high mediator concentrations (*m*-coumarate and *p*-coumarate), decolorization rates decreased due to the enzyme activity inhibition. The efficiency of laccase mediator systems in the decolorization reaction depends principally on the mediator concentrations and laccase activity used (Rodrıguez-Couto et al. 2005).

The feasibility of the laccase mediator systems in biotransformation reactions depends on redox reversibility of the radical–substrate reaction, as well as on the balance between the stability and reactivity of the mediator radical which, in addition, should not inhibit enzyme activity (Camarero et al. 2005).

Despite the efficiency of acetosyringone in Acid Orange 51 decolorization, this laccase mediator has two major drawbacks, namely high cost and the appearance of red-colored products. For this reason, HBT which gave similar decolorization rates to that of acetosyringone will be used for further work because of its low cost.

### Effect of pH and temperature on textile dye decolorization by laccase–HBT system

**Fig. 2:**
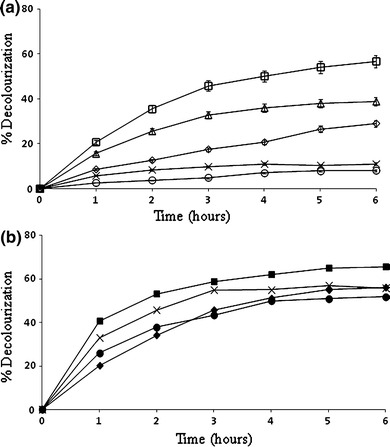
Effect of pH and temperature on textile dye decolorization by the*T*. *trogii* laccase–HBT system. **a** pH values: 4 (*open diamond*), 5 (*open square*), 6 (*open triangle*),7 (*multi symbol*), and 8 (*open circle*). **b** Temperature values (°C): 30 (*filled diamond*), 40 (*filled circle*), 50 (*filled square*) and 60 (*multi symbol*)

The decolorization of Acid Orange 51 occurred at pH 4.0, 5.0 and 6.0, with an optimum at pH 5.0 (Fig. 2a). No decolorization was observed at pH 8.0, although slow decolorization occurred at pH 7.0. These results indicated that the pH optimum for this laccase was substrate dependent, and purified laccases showed optimum pH values, estimated in 100 mM tartrate buffer, at 2.5 and 3 for oxidation of DMP (Zouari-Mechichi et al. 2006). However, the laccases were stable only at neutral pH values. Enzyme activity at higher pH decreased by the binding of a hydroxide anion to the T2/T3 coppers of laccase, interrupting internal electron transfer from the T1 to the T2/T3 centers (Munoz et al. 1997).

The stability of fungal laccases is generally higher at acidic pH (Leonowicz et al. 1984), although exceptions occur (Baldrian 2004) (the redox potential of HBT is ~1,100 mV at pH 4.0) (Xu et al. 2000). In addition, Xu et al. reported that there was no substantial protonation or deprotonation of HBT at pHs ranging between 4.0 and 9.0. In this pH range, therefore, the redox potential of HBT does not significantly depend on pH. The high and fairly constant value of *E*_0_ suggests that over a wide range of pHs, the reaction between *T*. *versicolor* laccase and HBT would be the least favorable compared to reactions of laccase with the other mediators. Additionally, in the pH range from 4 to 10, HBT generates a very unstable nitroxyl radical, –N(O·)–, which decays rapidly into species that are no longer functional as mediators (Potthast et al. 2001) (this process would also limit the effectiveness of HBT as a mediator).

Literature studies show that laccase-catalyzed dye oxidation is affected by the temperature (Nyanhongo et al. 2002). From the results presented in Fig. 2b, it can be concluded that the optimal temperature for dye decolorization was 50 °C; therefore, temperature variation in the range 30–50 °C did not seem to play an important role in the decolorization of AO51. Partial characterization of the laccase in the crude preparation showed an optimal pH at ~4. Activity was stable in the crude extract at room temperature, pH 7, for 24 h; more than 50 % activity was retained at pH 5. The laccase in the crude extract was also stable for 24 h at 50 °C; however, over 90 % activity was lost at 60 °C (Zouari-Mechichi et al. 2006).

### Analysis of variance and validation of the models

To optimize AO51 decolorization, Box–Behnken full factorial design with four factors was chosen. Twenty-five different experiments and four replicates at the center point and thus a total of 29 experiments were employed in this study. The center point replicates were chosen to verify any change in the estimation procedure as a measure of precision property.

The levels of the factors (*A* enzyme concentration, *B* HBT concentration, *C* dye concentration and *D* incubation time) and the results from the 29 experiments are presented in Tables 2 and 3, respectively. The temperature and the pH were fixed at 30 °C and 5.0, respectively.

Results of experiments based on the Box–Behnken design were used to estimate the model coefficients.

The fitted model expressed in actual variables is represented by Eq. (1). 

The fit quality of the yield model was confirmed using the analysis of variance (ANOVA) (Table 4). The adjusted regression sum of squares value (*R*^2^) was 90 %. At the same time, a relatively low value of the coefficient of variation (CV = 12.33 %) indicates a better accuracy and reliability of experiments. *F*_exp_ = 9.24 > *F*_0,05_ (υ_x_ (14), υ_r_ (14)) = 2.48. This implies that the model is statistically significant at 95 %.

**Table 4:** Summary of analysis of variance results; ANOVA for response surface quadratic model of dye Acid Orange 51

Source of variation	Sum of squares	Degrees of freedom (υ)	Mean square	*F* value	*p* > *F*	Significance
Model	7,374.7	14	526.8	9.2	<0.0001*	Significant
Residuals	798.3	14	57.0			
LOF	790.2	10	79.0	39.4	0.0015*	Significant
Error	8.0	4	2.0			

* Statistically significant at 95 % confidence level

To ensure that the resulting model summarizes the experimental and the evolution of the response studied as a function of changes shift the factors studied, we made seven checkpoints (Table 5) located in the area of study and proposed by the software Design-Expert version 7.0 (STAT-EASE Inc., Minneapolis, USA).

**Table 5:** Seven checkpoint results

Run	*A*	*B*	*C*	*D*	*R*	*y*_i_			SD	dU	*t*_exp_	ddl	Significance (%)
1	−0.5	0.5	−0.5	−0.5	68.73	68.73	54.45	14.27	9.77	0.313	1.27	14	22
2	0.5	0.5	0.5	−0.5	64.58	64.58	63.26	1.32	9.77	0.313	0.12	14	91
3	0.5	−0.5	−0.5	0.5	71.23	71.23	66.81	4.42	9.77	0.313	0.39	14	70
4	0.5	0.5	0.5	0.5	73.07	73.07	63.26	9.81	9.77	0.312	0.88	14	40
5	−0.5	−0.5	0.5	−0.5	56.71	56.71	45.82	10.89	9.77	0.313	0.97	14	35
6	−0.5	0.5	−0.5	0.5	69.74	69.74	59.53	10.21	9.77	0.312	0.91	14	38
7	−0.5	−0.5	0.5	0.5	60.44	60.44	54.25	6.19	9.77	0.312	0.55	14	59

*A* enzyme concentration, *B* HBT concentration, *C* dye concentration, *D* incubation time, *R* response

The seven checkpoint results were used to validate the fitted model. Table 5 shows that the measured values (*y*_i_) were close to those calculated () using the model equation. In addition, the differences between calculated and measured responses were not significant (*t* test; *p* < 95 %).

*t*_exp_ < *t*_crit(_υ_r = 14; 0.05)_ = 2.145 where *t*_exp_ value is below the critical value of Student’s (*t*_crit_) at 95 % for (υ_r_) degrees of freedom.

Thus, the results obtained (Tables 3, 4) confirm the validity of the Acid Orange 51 model, which was adequate to describe the response surfaces, and it can be used as a prediction equation in the design space.

**Fig. 3:**
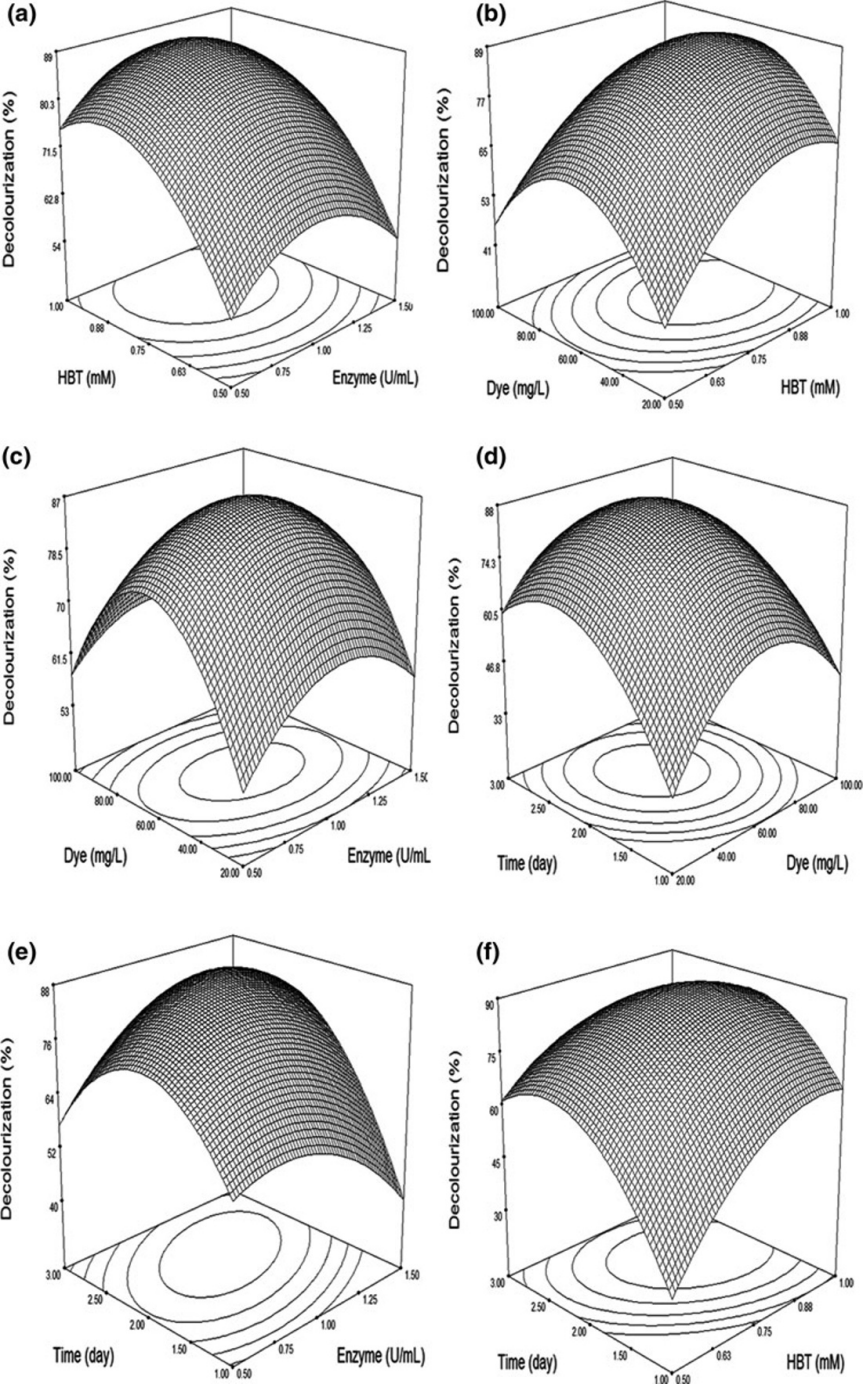
3D surface plot for the removal of Acid Orange 51 by crude *T. trogii* laccase as a function of **a** HBT and enzyme concentration, **b** dye concentration and HBT concentration, **c** enzyme concentration and dye concentration, **d** dye concentration and incubation time, **e** enzyme concentration and incubation time, **f** HBT concentration and incubation time

The relationships between reaction factors and response can be better understood by examining the planned series of contour plots (Fig. 3a–f), which were generated from the predictive model described above.

### Graphical interpretation of the response surface model

The response surface curves are plotted to explain the interaction of the variables and to determine the optimum level of each variable to reach a maximum decolorization yield. The response surfaces curves are presented in Fig. 3a–f obtained at a fixed temperature of decolorization (30 °C) and pH 5.0. Each figure demonstrates the effect of two factors, while the others were fixed at zero level.

As can be seen in Fig. 1, HBT redox mediator plays a key role in the decolorization of AO51 and is one of the most efficient mediators for laccases activity. It enhances the rate of laccase-mediated dye degradation, bleaching of pulp and other environmental pollutants (Murugesan et al. 2007; Gracia et al. 2003; Khlifi et al. 2010).

Figure 3a represents the effect of varying enzyme and HBT concentrations at fixed levels of incubation time and dye. The response plot revealed that an increase in enzyme concentration increased the decolorization level. The rate of decolorization increased with the increase in HBT concentration; however, at lower enzyme concentration (>1.0 U/mL), HBT started to inhibit the decolorization rate at the concentration above 1.2 mM suggesting that HBT was toxic to laccase beyond this concentration. Maximum dye decolorization of 86.1 ± 4.42 % occurred at 0.75 mM HBT with 60 mg/L dye. Similar results were observed in other studies (Murugesan et al. 2007). The addition of mediators could improve azo dye decolorization (Rodrıguez-Couto et al. 2005).

The results presented in Fig. 3e indicate that the variation of enzyme concentration has only a weak effect on AO51 decolorization rate. In fact, the center value attributed to the enzyme concentration gives the maximum decolorization (87.80 %). From both sides of this value, color removal does not exceed 75 %. This result is consistent with that of Tavares et al. (2009).

**Fig. 4:**
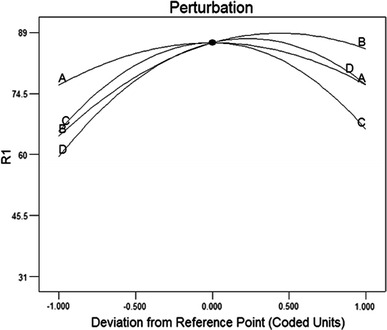
Perturbation graph showing the effect of each of the independent variables on Acid Orange 51 decolorization while keeping other variables at their respective mid-point levels. (*A*) Enzyme concentration, (*B*) HBT concentration, dye concentration (*C*) and (*D*) reaction time

The stability of the enzyme over a period of time and its concentration are more important for enzymatic dye decolorization. Figure 3e represents the effect of varying concentrations of enzyme at different incubation times on AO51 decolorization under 60 mg/L dye and 0.7 mM HBT concentrations. The results indicate that the response increased on increasing the enzyme concentration as well as the incubation time (more than 74.0 %). The best decolorization value of 87.90 % was observed on a surface plot at 2 days with 1 U/mL enzyme; however, AO51 decolorization increased when the concentration of the enzyme was increased by more than 1 U/mL (Fig. 4). Our results indicate that the laccase from *Trametes.* sp is stable over 48 h at the conditions employed. Many laccases have been identified to be thermostable and are even more active even under high stress conditions (Niku-Paavola et al., 2004; Zouari-Mechichi et al. 2006).

### Optimization

An optimization process was carried out to determine the optimum decolorization condition of acid Orange 51 (AO51) using the Design-Expert version 7.0 (STAT-EASE Inc., Minneapolis, USA) software.

The maximum predicted AO51 decolorization was 87.35 ± 4.42 % at an enzyme concentration of 0.89 U/mL, HBT concentration of 0.79 mM and dye concentration of 60.38 mg/L. Then, the efficiency of prediction of the polynomial model was tested by performing the experiment under the predicted optimal condition. The value of predicted decolorization yields was in good agreement with the experimental value, which indicated that the developed model was quite efficient and adequate.

According to the Design-Expert version 7.0 software optimization step, the desired goal for each operational condition (dye concentration) was chosen “within the range”, while enzyme concentration, HBT concentration and incubation time were defined “as minimum” to achieve the highest decolorization yields and minimize the process cost simultaneously.

The program combines the individual desirabilities into a single number and then searches to maximize this function. Accordingly, the optimum working conditions and respective yield decolorization were established. As seen in Tables 1, 5 and 6 solutions were found with desirability range from 0.58 and 0.75, respectively.

**Table 6:** Optimization

Constraints				
Name	Goal	Lower limit	Upper limit	Lower weight
Enzyme (U/mL)	Minimize	−1	1	1
HBT (mM)	Minimize	−1	1	1
Dye (mg/L)	Is in range	−1	1	1
Time (days)	Minimize	−1	1	1
Decolorization (%)	Maximize	31.02	87.98	10

Number	Enzyme (U/mL)	HBT (mM)	Dye (mg/L)	Time (Days)	Decolorization (%)	Desirability
1	0.89	0.79	60.38	1.99	87.35	0.58

Constraints				
Name	Goal	Lower limit	Upper limit	Lower weight
Enzyme (U/mL)	Minimize	−1	1	1
HBT (mM)	Minimize	−1	1	1
Dye (mg/L)	Is in range	−1	1	1
Time (days)	Minimize	−1	1	1
Decolorization (%)	Maximize	31.02	87.98	1

Number	Enzyme (U/mL)	HBT (mM)	Dye (mg/L)	Time (Days)	Decolorization (%)	Desirability
1	0.5	0.65	65.62	1.46	64.14	0.75
2	0.5	0.65	65.33	1.46	64.11	0.75
3	0.5	0.65	65.76	1.47	64.09	0.75
4	0.54	0.64	65.09	1.47	64.94	0.75
5	0.5	0.65	63.78	1.55	65.88	0.75

The optimal values of the four variables predicted by the model using a global desirability function (0.75) were: dye range of 63.78–65.76 mg/L, enzyme concentration of 0.5 U/mL, HBT concentration of 0.64 mM and incubation time of 1.5 day. The maximum predicted decolorization yield was 87.98 % for AO51.

### Phytotoxicity assay

Phytotoxicity assays were performed using laccase-treated and untreated dye. Crude laccase preparation obtained from Cu-induced cultures of *T*. *trogii* in solid culture with sawdust as support substrate was used for AO51 decolorization. The germination index was used as an indicator of phytotoxicity in Petri dishes. To avoid pH effect on germination index, the pHs of all reaction mixtures were adjusted to pH 8.0 at the end.

**Fig. 5:**
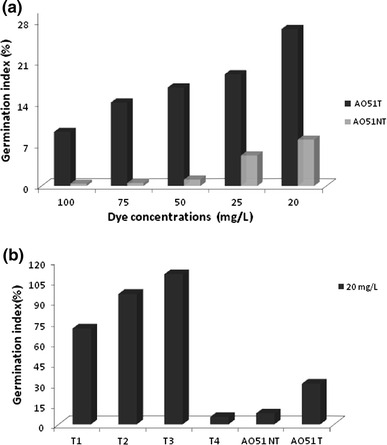
Germination index of Acid Orange 51(AO51) before and after decolorization by crude laccase preparation at **a** 20 mg/L, **b** different dye concentrations 100-75-50-25 and 20 mg/L. *T1* enzyme with HBT, *T2* enzyme without HBT and *T3* heat-killed enzymes, *T4* enzyme without HBT with AO51

Figure 5a shows that laccase-untreated AO51 is toxic compared to the zero germination index, whereas with laccase-treated AO51, the germination index for 100, 75 and 20 mg/L dye concentrations were 8.8, 10.8 and 29.0 %, respectively.

The acid dye toxicity to seed germination may be due to aromatic amines reaction products produced during the dyeing processes. Phytotoxicity was inversely proportional to dye concentration. The maximum germination index (29.0 %) was shown at 20 mg/L dye concentration. However in the presence of HBT, the phytotoxicity of the treated dye was increased (Fig. 5b).

*T. trogii* crude laccase treatment was efficient at decolorizing the AO51 and able to detoxify this dye class (as determined by the germination index assay and shown in Fig. 5), while the use of HBT as laccase mediator system gave more efficiently decolorized solutions of azo dye but decreased their germination index. These results demonstrate the potential and limitations of using crude laccase preparation to enzymatically decolorize acid azo dye classes and reduce dye toxicity in a single step.

In the same context, anaerobic bacteria decolorization of azo dyes may generate toxic aromatic amines (reviewed in Delée et al. 1998; Kaushik and Malik 2009) which can be detoxified by a subsequent aerobic bacterial step (Gottlieb et al. 2003). However, using white rot fungi potentially represents a single step to decolorize and detoxify. Furthermore using purified enzymes will eliminate fungal metabolites which have been shown to contribute to toxicity (Ramsay and Nguyen 2002), and any toxicity changes would be due to dye removal and/or decolorized products.

## Conclusions

By using cheap agro-industrial wastes, such as sawdust, a potential laccase was obtained. The low cost of laccase production may further broaden its application in textile wastewater treatment.

Box–Behnken experimental design and RSM are important tools to optimize the conditions for the decolorization of synthetic dyes, reduce the number of experiments required to optimize the system and provide useful information concerning the effect of different conditions and possible interactions. The models employed provided a good predictive accuracy for the variables tested in terms of effective dye decolorization and correlation coefficients (*R*^2^) of 0.9, for the degradation of AO51. Using the model, therefore, the degradative response for the variables tested can be predicted at any point in the system.

The overall results show that laccase from *T. trogii* produced under sawdust in solid culture has great possibilities to decolorize the textile dyes present in the effluents of textile industries; however, further reactor-scale studies are required for actual industrial applications.

## Electronic supplementary material

Below is the link to the electronic supplementary material.

Supplementary material 1 (TIFF 386 kb)
