# Supplementary figures and images for: Decolorization of the azo dye Acid Orange 51 by laccase produced in solid culture of a newly isolated Trametes trogii strain
Source: 3 Biotech. 2012 Jul 17;3(2):115–25. doi: 10.1007/s13205-012-0076-2 (PMC3597134; doi:10.1007/s13205-012-0076-2)

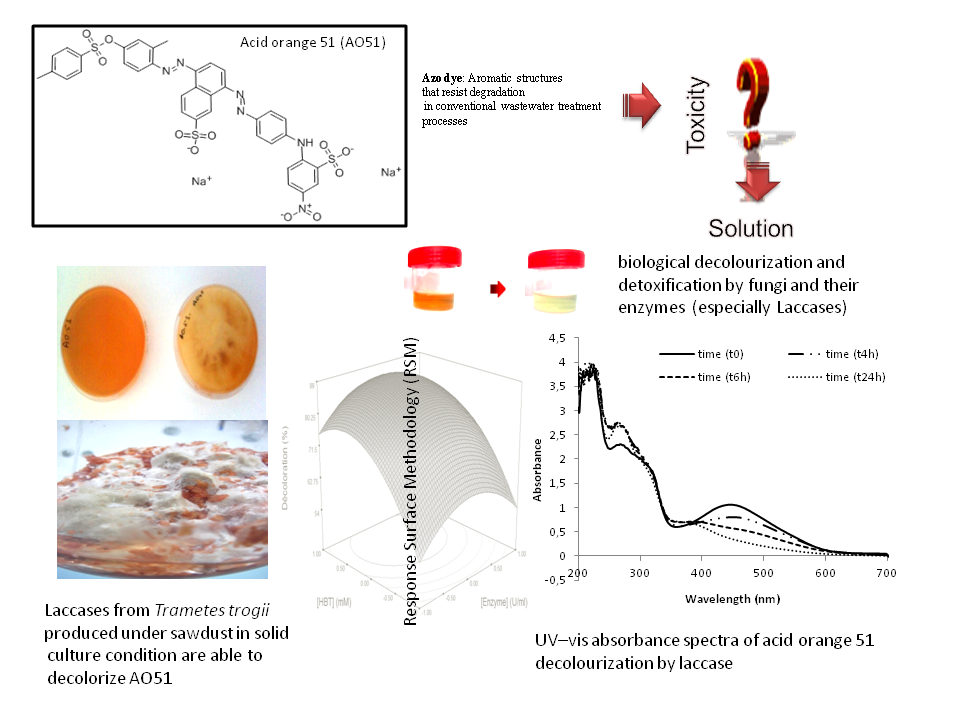

Supplement: Supplementary file 1 — Supplementary material 1 (TIFF 386 kb) [file 13205_2012_76_MOESM1_ESM.tif]
